# Organic fluorescent probes for live-cell super-resolution imaging

**DOI:** 10.1007/s12200-023-00090-3

**Published:** 2023-11-10

**Authors:** Xinxin Duan, Meng Zhang, Yu-Hui Zhang

**Affiliations:** Britton Chance Center for Biomedical Photonics, MoE Key Laboratory for Biomedical Photonics, Advanced Biomedical Imaging Facility-Wuhan National Laboratory for Optoelectronics, Huazhong University of Science and Technology, Wuhan, 430074 China

**Keywords:** Super-resolution imaging, Organic fluorescent dyes, Live-cell imaging, Cell-impermeable organic probes

## Abstract

The development of super-resolution technology has made it possible to investigate the ultrastructure of intracellular organelles by fluorescence microscopy, which has greatly facilitated the development of life sciences and biomedicine. To realize super-resolution imaging of living cells, both advanced imaging systems and excellent fluorescent probes are required. Traditional fluorescent probes have good availability, but that is not the case for probes for live-cell super-resolution imaging. In this review, we first introduce the principles of various super-resolution technologies and their probe requirements, then summarize the existing designs and delivery strategies of super-resolution probes for live-cell imaging, and finally provide a brief conclusion and overview of the future.

**Graphical Abstract:**

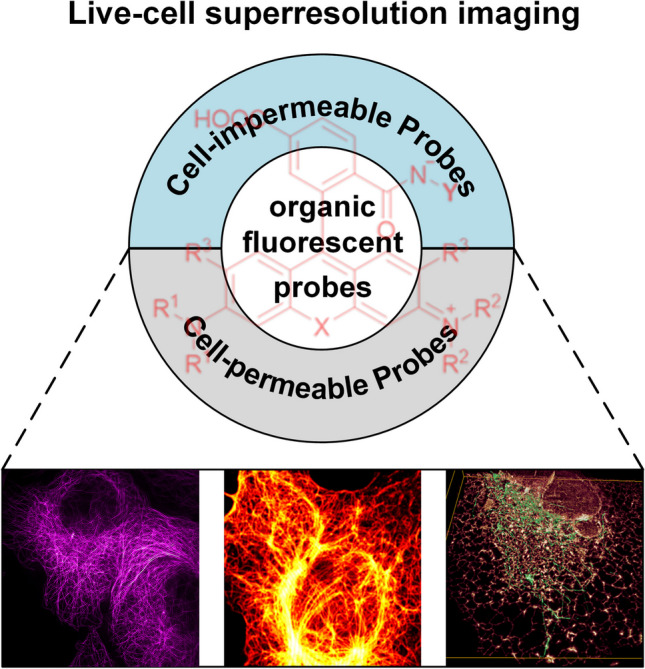

## Introduction

The cell is like a complex factory, and the various subcellular structures, organelles, are like precision instruments that have different functions and that are constantly working together to maintain its normal physiologic activities [1–3]. Abnormalities in subcellular structures may lead to changes in cellular physiologic functions, which in turn can lead to various diseases [4–6]. Currently, many diseases such as cancer, cardiovascular diseases, and neurodegenerative diseases, are found to be related to dysfunction of cellular organelles [7–9]. Therefore, the study of the fine structure and function of these subcellular structures is of great significance to the development of biomedicine.

Microscopy, as a powerful tool for investigating cellular structure and function, plays an important role in promoting the development of cell biology and biomedicine [10]. Since the first microscope was developed by Janssen at the end of the sixteenth century, optical microscopes have been widely used in various fields of life sciences and biomedicine, and microscopes have become indispensable research tools [10, 11]. In particular, the development of fluorescence microscopic imaging techniques in combination with fluorescent dyes has enabled the visualization of biomolecules in cells and tissues, revealing their accurate locations, dynamics and interactions [10, 12, 13]. The advent of fluorescence microscopy has significantly expanded researchers’ understanding of the structure and function of living organisms. However, due to the optical diffraction limit, the lateral resolution of traditional fluorescence microscopes can only reach minima of 200 to 300 nm, and the axial resolution can only reach 500 nm [10, 12, 13]. While the physical dimensions of many subcellular structures within the cell, as well as their interaction sites, are smaller than 200 nm, making it difficult to study the subcellular structures and their interactions in detail using traditional fluorescence microscopes [14, 15]. This has significantly limited the progress of cell biology and medicine. The advent of scanning electron microscopy and transmission electron microscopy has made it possible to observe intracellular ultrastructures in fine detail. However, electron-based microscopes require complicated sample preparation, and the imaging conditions are too harsh to be used for observing living cells [16, 17], so although they can break the optical diffraction limit, they cannot be used to study the dynamic changes of subcellular structures in living cells.

Over the past few decades, researchers have overcome the limitations of optical diffraction by combining physical, chemical, biological, and other approaches and developed a series of super-resolution fluorescence imaging techniques, which have made it possible to use fluorescence microscopy to observe the dynamic changes of various subcellular structures in living cells [18–23]. Since then, super-resolution imaging techniques have been widely used to observe subcellular structure and dynamic processes, and have provided many new insights into cell biology and biomedicine [12]. However, achieving super-resolution imaging of living cells requires fluorescent probes with excellent optical properties to label various subcellular structures [10, 15]. The commonly used fluorescent probes can be mainly classified into fluorescent proteins and organic fluorescent dyes [24, 25]. Compared to fluorescent proteins, organic fluorescent dyes have various advantages, such as superior fluorescence brightness and better photostability, making them more suitable for super-resolution imaging [26–28]. In this review, we will provide a concise overview of the principles of common super-resolution imaging systems and their requirements for fluorescent probes. In addition, we will focus on the fluorescent dyes that have been developed in the past few years and that can be used for super-resolution imaging of living cells. This will provide a reference for researchers for performing super-resolution imaging of living cells.

## Principles of super-resolution technologies and their requirements for fluorescent probes

### Principles of SMLM technology and its requirements for fluorescent probes

**Fig. 1:**
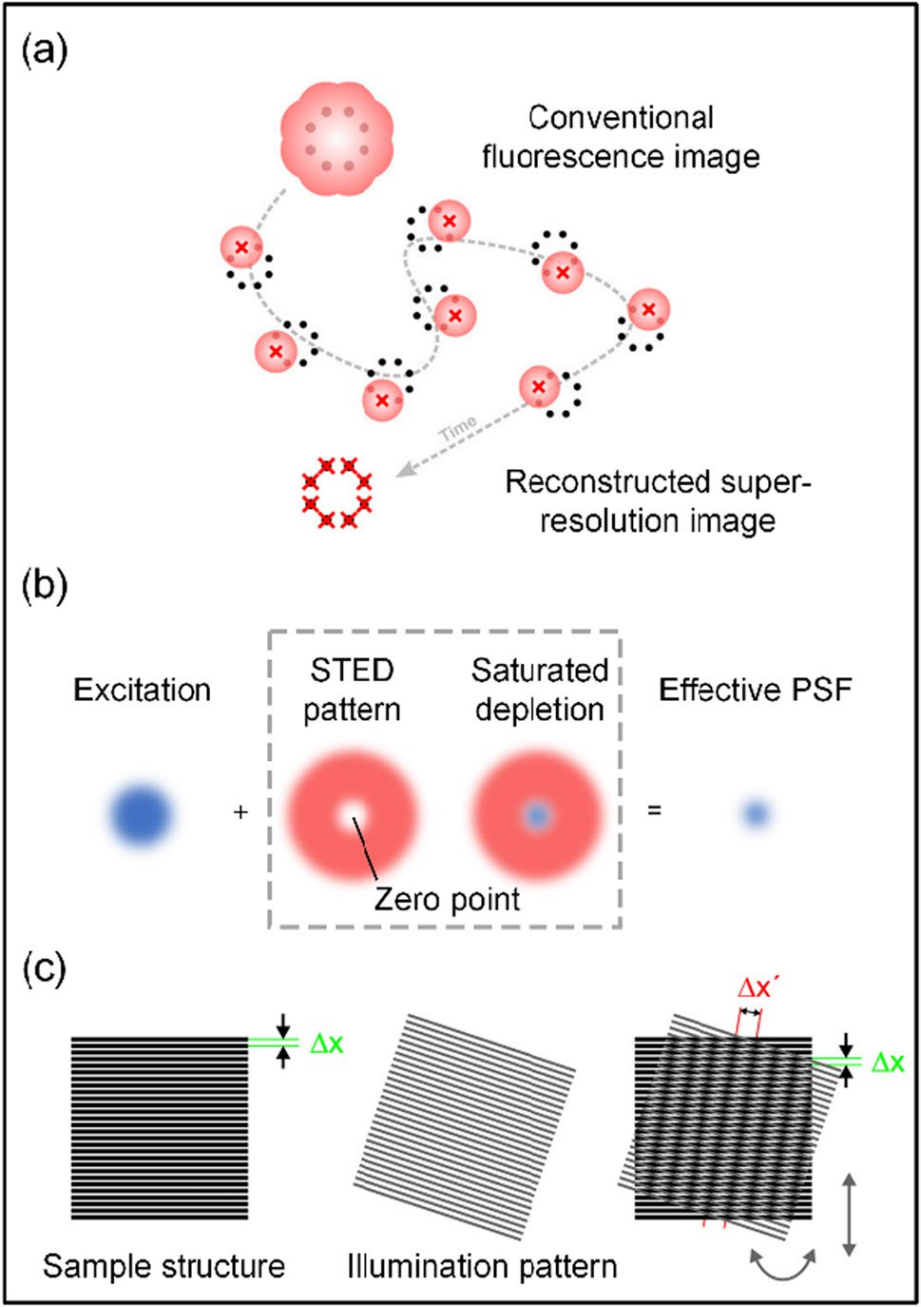
**a** Principles of SMLM technology. Reprinted (adapted) with permission from Ref. [29]. **b** Principles of STED technology. The blue point represents the dyes excited by excitation beam and the red doughnut represents the depletion beam. **c** Principles of SIM technology. Reprinted (adapted) with permission from Ref. [30]

Single-molecule localization microscopy (SMLM) is an advanced microscopy technique capable of achieving imaging beyond the resolution of traditional optical microscopy in cells and biological samples. SMLM includes photoactivated localization microscopy (PALM) and stochastic optical reconstruction microscopy (STORM), and the basic principles of PALM and STORM are similar [22, 23]. The main difference between them is that PALM uses fluorescent proteins to label intracellular molecules while STORM uses organic fluorescent dyes. Single molecule localization microscopy (SMLM) exploits the intermittent blinking behavior of fluorescent molecules to achieve improved imaging resolution (Fig. 1a). During the imaging process, the activation of fluorescent molecules is controlled to alternate between “on” and “off” states. This ensures that only a limited number of molecules are photoactivated in each image, reducing the potential for interference between fluorescent spots. The intensity distribution of each spot is then analyzed using methods such as Gaussian fitting. This allows the central position of each fluorescent molecule to be more precisely determined. The process is repeated thousands of times during image acquisition, meticulously compiling the positions of fluorescent molecules. This approach sacrifices temporal resolution in exchange for spatial resolution, allowing localization accuracy to be improved to a few nanometers.

In summary, for live-cell single-molecule super-resolution imaging systems, fluorescent molecules must first be capable of labeling live cells. Additionally, they should exhibit “on–off” behavior, while individual fluorescent molecules should have sufficiently high brightness to ensure accurate detection and localization in the image. These fluorescent moieties must also have good photostability to undergo a sufficient number of on–off cycles thus ensuring the detection of a significant number of individual molecules [28, 29].

### Principles of STED technology and its requirements for fluorescent probes

Stimulated emission depletion microscopy (STED) is a breakthrough super-resolution microscopy technique that uses a stimulated emission phenomenon to deplete the fluorescence signal and achieve imaging beyond the resolution of conventional fluorescence microscope [18, 19]. Conventional STED imaging systems require two laser beams to illuminate the sample simultaneously (Fig. 1b). The first beam, called the excitation beam, is used to excite fluorescent molecules to emit fluorescence signals. The second beam, the “depletion beam”, creates an intensity distribution at specific doughnut-shaped locations and returns the excited fluorescent molecules from the excited state to the ground state. When excited fluorescent molecules are exposed to the depletion beam, they undergo a stimulated emission process that results in a reduction of the emitted fluorescence. This causes only the fluorescent molecules within the intensity distribution of the depletion beam to emit a signal, while other regions are suppressed due to stimulated emission. By controlling the relative intensities and phases of the excitation and depletion beams, precise control of the fluorescence signal can be achieved.

Live cell STED imaging requires fluorescent molecules that not only label the cells but also have specific properties. The emission peak of the fluorescent dyes should match the wavelengths of the excitation and depletion beams to ensure that the effect is maximized. In addition, the fluorescent dyes must have excellent fluorescence brightness and photostability to ensure that they can undergo multiple cycles of excitation and depletion without significant photobleaching.

### Principles of SIM technology and its requirements for fluorescent probes

Traditional optical microscopy systems are usually only capable of receiving low-frequency information, which results in the loss of high-frequency information about the fine structure of the samples, thus limiting the resolution of the optical imaging system [31]. Structured Illumination Microscopy (SIM) technology combines low-frequency information acquired by imaging with illuminated structured light of a known spatial distribution function to compute high-frequency information about the fine structure of the sample, thereby enhancing the resolution of the optical imaging system. (Fig. 1c). Although the resolution improvement achieved by the SIM technique is relatively small compared with that achieved by the two aforementioned super-resolution imaging methods, usually reaching only about 100 nm, it is preferred by biologists because of its lower excitation light intensity and faster imaging speed, which is more suitable for live cell imaging.

In recent years, with the continuous development of SIM imaging methods, the resolution limit has reached 60 nm and the imaging speed has been further improved. The application of SIM technology in the life science field has thus become more and more widespread [32, 33]. Although SIM technology itself has no special requirements for fluorescent groups, its imaging principle requires the reconstruction of nine original images to generate one super-resolution image, which requires nine times more data to be acquired compared with traditional fluorescence microscopy. Therefore, in order to realize fast and long-term SIM imaging of subcellular structures of living cells, higher demands are placed on the anti-photobleaching ability and fluorescence brightness of fluorescent molecules.

## Organic fluorescent probes for live-cell super-resolution imaging

### Recognition groups for organic fluorescent probes

**Fig.2:**
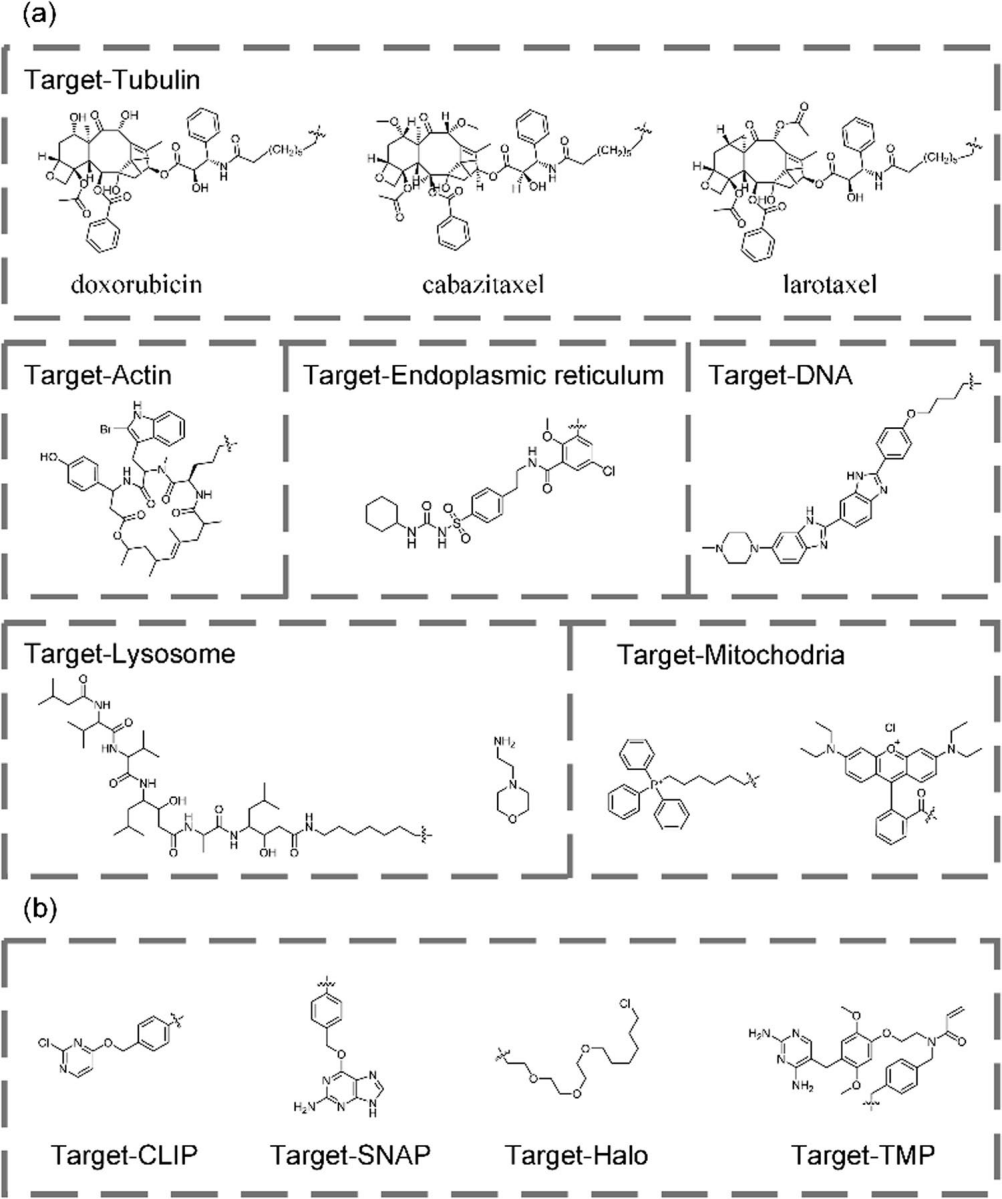
**a** Commonly used chemical recognition groups for various subcellular structures. **b** Chemical structures of SNAP, CLIP, Halo and TMP substrates

The properties of the fluorescent dyes of the fluorescent probes have a significant impact on the final results of super-resolution imaging. Compared to fluorescent proteins, organic fluorescent dyes have a number of advantages such as: high fluorescence intensity, small physical dimensions, high photostability, and are easy to modify [26–28]. Therefore, organic fluorescent dyes are more suitable for super-resolution imaging. However, unlike fluorescent proteins, organic fluorescent dyes usually cannot specifically target different biomolecules and usually need to be covalently linked to specific recognition groups to achieve specific labeling. Figure 2a summarizes the commonly used recognition groups for labeling subcellular structures of living cells. Although specific labeling of some subcellular structures (such as tubulin, actin, and mitochondria) can be achieved by covalently linking the above recognition groups (Fig. 2a) to form organic fluorescent probes, the quantity of these recognition groups remains relatively small compared to the vast number of proteins present in a cell, thus limiting the application of organic fluorescent dyes in cell biology [34–38]. For microtubule labeling, doxorubicin, cabazitaxel and larotaxel are the most commonly used probe recognition groups [39]. Cabazitaxel provides the best microtubule labeling effect, compared to those of docetaxel and larotaxel, but it is also more cytotoxic. For live cell actin labeling, organic probes are primarily based on jasplakinolide as the recognition group [40]. To overcome the problems of poor membrane permeability and inhomogeneous staining associated with jasplakinolide-based probes, researchers have discovered that the 6'-carboxy-carbopyronine scaffold is much less susceptible to efflux and allows for efficient staining without the need for efflux pump inhibitors. Although other recognition groups for actin labeling in living cells have been reported in recent years, their specificity needs to be further verified [41]. For mitochondrial labeling in living cells, triphenylphosphine is the most commonly used recognition group, but positively charged fluorophores such as Rhodamine B, Cy3, and Cy5 can also be used for this.

To take advantage of organic fluorescent dyes to label various proteins in cells similar to fluorescent proteins, Johnsson et al. developed the SNAP-tag labeling technology in 2003 by modifying O^6^-alkylguanine DNA alkyltransferase [42]. In 2008, Gautier and colleagues developed the AGT-based CLIP-tag that reacts specifically with O^2^-benzylcytosine derivates [43]. Similarly, there are other tagging techniques such as Halo-tag [44], and TMP-tag [45, 46]. These protein labeling techniques solve the problem of lacking specificity of organic fluorescent dyes and greatly expand the application of fluorescent probes in life sciences (Fig. 2b).

### Cell-permeable organic fluorescent probes for live-cell super-resolution imaging

**Fig.3:**
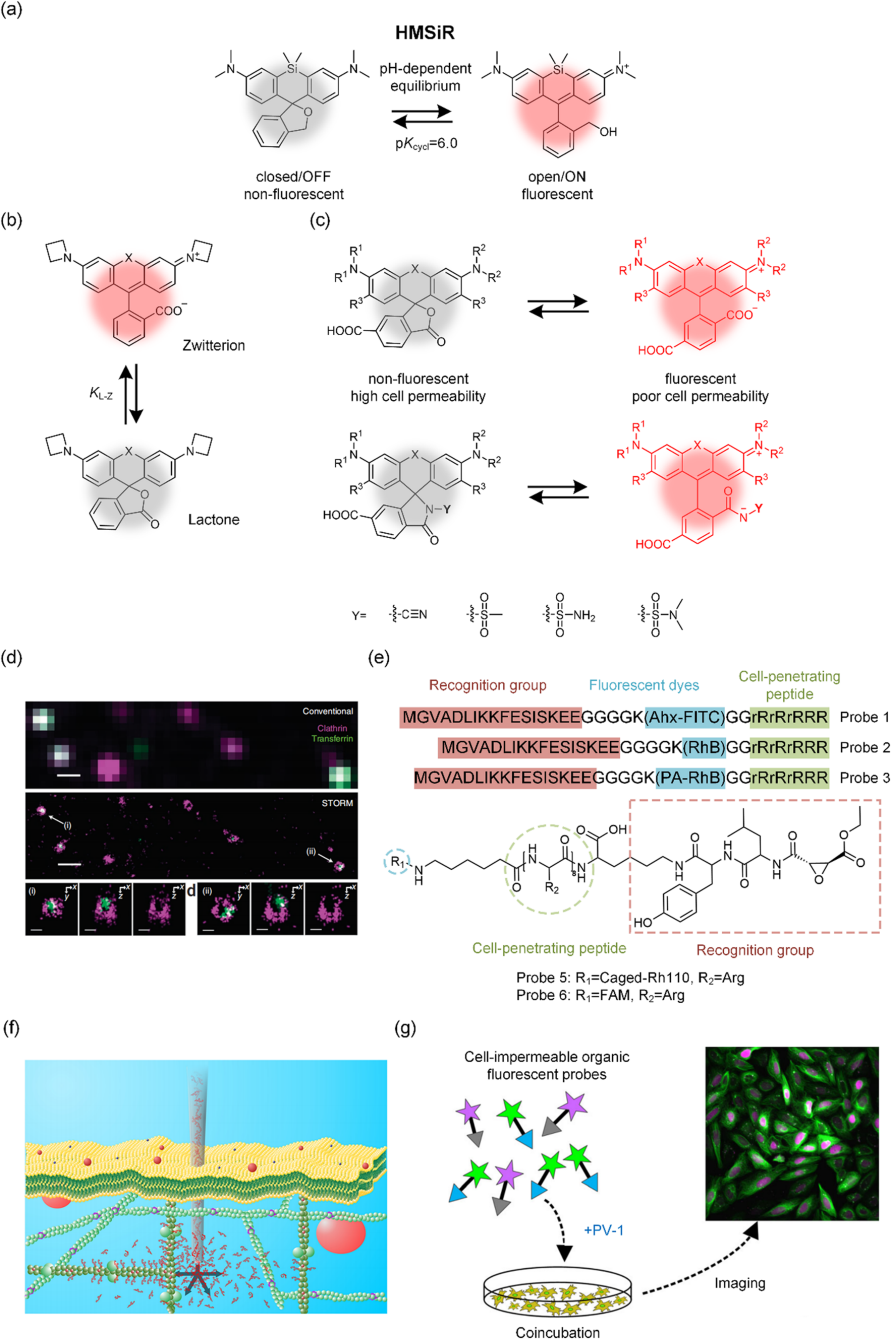
**a** On–off principle of HMSiR. **b** Principle of lactone-zwitterion equilibrium constant (K_L-Z_) theory. **c** Mechanism of improved cell-permeability and fluorogenicity of rhodamine derivatives. **d** Delivering SNAP-Alexa 647 into live cells for SMLM imaging using bead-loading method. Reprinted (adapted) with permission from [88]. **e** Covalent conjugation of cell-penetrating peptide with probe for live cell delivery. **f** Principle of nanoinjection. Reprinted (adapted) with permission from [91]. **g** Schematic showing PV-1 delivering cell-impermeable probes into living cells. Reprinted (adapted) with permission from Ref. [94]

Due to the selective permeability of cell membranes, not all fluorescent dyes are able to enter living cells. Here, we review the available fluorescent dyes suitable for super-resolution imaging of living cells. For SMLM imaging, a spontaneously blinking fluorophore called HM-SiR (Fig. 3a), which is based on an intramolecular spirocyclization reaction, was developed by Shin-Nosuke Uno et al. in 2014 [47]. This fluorescent dye enables spontaneous blinking for live-cell SMLM under physiologic conditions, regardless of laser irradiation intensity or thiol concentration. In 2018, Patrick J. Macdonald et al. reported a spontaneously blinking yellow dye called FRD. Regrettably, the slow blinking rate of FRD, ranging from seconds to minutes, poses a limitation on its effectiveness for some fast dynamic imaging applications [48]. In 2019, Juan Tang et al. introduced a thiocarbonyl group and discovered that fluorophores undergo photoinduced electron transfer (PET)-induced fluorescence quenching. This quenching can be reversed by oxidative desulfurization upon exposure to air and visible light that is within their absorption range. Based on the above principles, they have developed probes, such as SDMAP, that can be activated by visible light, thereby effectively mitigating the phototoxicity associated with activation by UV light [49]. The same year, based on the lactone-zwitterion equilibrium constant (KL-Z) theory [50–52] (Fig. 3b), Qinsi Zheng et al. developed the JF525 dye suitable for live cell imaging. They further introduced hydroxymethyl modifications to create a spontaneously blinking derivative known as HM-JF525 [53]. However, whether HM-JF525 can be used for live cell SMLM imaging needs to be further validated. In 2020, Weijie Chi et al. proposed a unified push–pull model based on the properties of 24 representative rhodamine dyes in understanding and designing the fluorescence properties of rhodamine dyes [54, 55]. A spontaneously blinking dye HM-DS655, which can be used for SMLM imaging of living cells, was designed based on the above theory [55]. In 2021, Jonathan Tyson et al. reported a spontaneously blinking near infrared (NIR) rhodamine dye, Yale676sb, which can be used for SMLM imaging of living cells [56]. Two-color SMLM imaging of live cells has been achieved by using the above-mentioned dye in combination with HM-SIR. Lushun Wang et al. found that when irradiated with light in the presence of air, the oxime-capped fluorophores undergo their carbonyl derivatives, leading to the revival of robust fluorophore fluorescence [57]. Based on this principle, they developed a series of on–off probes suitable for SMLM imaging of living cells. In 2023, Ying Zheng et al. designed and synthesized two spontaneously blinking sulfonamide-rhodamine dyes, STMR and SRhB, both suitable for live-cell SMLM imaging. And they found that STMR, characterized by high emission rates, is well suited for imaging dynamic structures, while SRhB, with prolonged on times and enhanced photostability, proved effective for imaging relatively “static” nuclei and microtubules [58, 59].

Fluorescent probes used for STED imaging of living cells also have stringent requirements; they must not only have excellent photostability and membrane permeability, but also be able to transition from the excited state to the ground state under the depletion beam [18, 19, 60]. Rhodamine-based ATTO647N is an organic dye with a high fluorescence quantum yield and a large extinction cross-section. Due to these properties, it has been widely used as a probe for STED nanoscopy [61, 62]. However, ATTO647N binds unspecifically to the cell membrane, and this may have an effect on imaging results [63]. In 2012, Christian A. Wurm et al. developed new rhodamine-based red fluorophores Abberior STAR 635 based on KK114 [64, 65], which inherits the high photostability of KK114 and also has excellent membrane permeability that can be used for STED imaging of living cells [63]. In 2013, Grazvydas Lukinavičius et al. developed a highly permeable and biocompatible near-infrared silicon-rhodamine (SiR) fluorophore with the ability to enable STED imaging of living cellular microtubules [66]. Subsequent research has further demonstrated that SiR dyes are highly suitable for both single- and two-color live cell STED imaging [67]. In 2016, Alexey N. Butkevich et al. developed a series of novel rhodamine dyes, such as 510R and 580R, with excellent fluorescence brightness and photostability and good membrane permeability, suitable for multicolor STED imaging of living cells with 40–60 nm optical resolution [68]. In 2017, Grazvydas Lukinavičius et al. developed the SiR700, which combined with SiR enables two-color STED imaging of subcellular structures in living cells [69]. In 2020, Lu Wang and colleagues reported a novel strategy to manipulate the equilibrium between fluorescent zwitterions and non-fluorescent spirolactone forms. This was achieved by transforming the 2'-carboxyl group in rhodamines into electron-deficient amides. Importantly, this modification had remarkable potential to enhance both cell permeability and fluorogenicity of the dyes while having no discernible effect on the spectroscopic properties of rhodamine [70] (Fig. 3c). Based on the above theory, they synthesized a series of fluorescent dyes, including MaP555, MaP618, and MaP700. These dyes have demonstrated their applicability for STED live-cell imaging, marking a notable advance in the field of super-resolution microscopy [71]. To mediate the equilibrium between spirolactone and zwitterions without affecting rhodamine’s spectral properties, Jonas Bucevičius et al. utilized the neighboring group effect by producing 4'-positional isomers of carboxyl rhodamine. This theory has led to the development of excellent photostable dyes, including 4-580CP and 4-610CP, which are suitable for STED imaging of living cells [72]. Due to the challenges posed by existing rhodamine caging strategies, including water solubility and the potential formation of toxic byproducts upon photoactivation, Richard Lincoln et al. developed a class of functionalized xanthones that efficiently and cleanly convert to the corresponding dihydropyran-fused pyronine dyes upon laser excitation. Based on the above strategy they developed PaX_560_ and realized STED imaging of living cells [73]. Furthermore, in recent years, a series of mitochondrial probes suitable for live-cell STED imaging have been developed through strategies such as conjugation of cyclooctatetraene (COT) to a benzo-fused cyanine dye [74–77]. This advance has significantly expanded our understanding of mitochondrial ultrastructure by enabling live cell STED imaging.

It has been generally accepted that live-cell SIM imaging does not place any additional demands on the performance of fluorescent dyes beyond those of conventional live-cell fluorescence imaging. However, as SIM technology is increasingly applied to live-cell imaging, researchers are better understanding its requirements and have discovered that live-cell SIM imaging also requires high-performance fluorescent dyes. If the signal-to-noise ratio is not strong enough during SIM imaging, it will introduce significant artifacts and affect the resolution of the image [78, 79]. Typically, live-cell SIM imaging needs 9 − 15 raw images to reconstruct a single super-resolution image. Artifacts can appear in the reconstructed results if the structure of the sample changes significantly during acquisition of these images. To avoid these artifacts, shorter exposure times are needed, which in turn requires excellent fluorescence brightness of the fluorescent probe. In addition, live-cell SIM systems are often used for long-term dynamic imaging, which places higher demands on the photostability of the dye. Currently, the fluorescent dyes suitable for long-term super-resolution imaging with live-cell SIM are mainly SiR and JF dyes [80, 81].

### Cell-impermeable organic fluorescent probes for live-cell super-resolution imaging

Generally, probes with higher lipophilicity exhibit better membrane permeability, while those with higher water solubility tend to exhibit better fluorescence performance in aqueous environments. However, probes often struggle to find a balance between lipophilicity and water solubility. In the quest for improved cell-permeability, the fluorescent performance of probes is often compromised. Probes such as Alexa 488 and ATTO 488, possess excellent fluorescence photostability, water solubility, and fluorescence brightness [82–85]. However, most of them lack cell permeability on their own or lose cell permeability after conjugation to a recognition moiety for specific labeling. As a result, they can only be used to label fixed cells or the plasma membranes of living cells [86, 87].

To address the above issues, developing methods to support cell-impermeable organic fluorescent probes in living cells could expand the range of options available for super-resolution imaging probes. In 2011, Sara A. Jones et al. used the bead-loading method to deliver the cell-impermeable organic fluorescent probe BG-Alexa 647 to living cells and combined it with dSTORM imaging technology to achieve three-dimensional, super-resolution dynamic imaging of the translocation process of clathrin-coated pits (CCPs) [88] (Fig. 3d). In 2014, Deng Pan et al. covalently linked a special cell-penetrating peptide (rR)_3_R_2_ with cell-impermeable probes, allowing delivery of PA-RhB-Lifeact and Caged-Rh110-Epoxysuccinyl into living cells and achievement of specific labeling [89, 90] (Fig. 3e). Using the above probes in combination with SMLM, Deng et al. observed the fine process of dynamic F-Actin reorganization in living cells. In 2015, Simon Hennig et al. proposed a nanopipette-assisted electrophoretic delivery strategy and successfully delivered ATTO 655-phalloidin into living cells. Using the above method in combination with the dSTORM system, super-resolution imaging of actin in living cells was achieved [91] (Fig. 3f). Other physical methods, such as electroporation, laser-induced photoporation, micro- and nanoinjection, micro- and nanostructure-mediated membrane disruption, and the emerging utilization of nanomachines or nanomotors, have been tested on conventional fluorescence microscopes. These methods may be employed in the future for super-resolution imaging of live cells [92]. In 2017, Yubing Han et al. further optimized the delivery strategy proposed by Pan and delivered various cell-impermeable probes, such as Lysosome-Alexa Fluor 647, Lysosome-ATTO 565, and Lysosome-ATTO 488, into living cells using cell-penetrating peptides. Using the above probes in combination with the SIM imaging system, long-term imaging of fine lysosome-mitochondrion interactions revealed four types of physical lysosome-mitochondrion interactions [93]. Using the aforementioned probes in conjunction with the SIM imaging system, long-term imaging of the intricate interactions between lysosomes and mitochondria revealed four types of physical interactions between them. In 2019, Meng Zhang et al. developed a simple and effective method to deliver cell-impermeable fluorescent probes into live cells using a cell-penetrating peptide, PV-1 [94] (Fig. 3g). Without covalently linking cell-penetrating peptides to probes, by simple co-incubation with PV-1, 22 different cell-impermeable organic fluorescent probes, including Tubulin-ATTO 488, SNAP-Alexa Flour 488, CLIP-Dy 547, Hoechst-Alexa Fluor 647, were efficiently delivered into living cells and specifically labeled a variety of organelles. Based on the above method, they obtained multi-color, long-term, live-cell SIM images of different organelles and revealed the dynamic interactions between various such subcellular structures. In 2022, they delivered Tubulin-ATTO 488 into live cells with the help of the above method, and by combining that with the IDDR-SPIM imaging system, they realized the three-dimensional dynamic visualization study of microtubules in live cells [95]. Furthermore, recent studies have shown that some cell-impermeable dyes, such as Rho565, can achieve live-cell labeling when conjugated to CA (the recognition groups for Halo-tag), but their permeability mechanism requires further investigation [96].

## Conclusion

In conclusion, fluorescent dyes are indispensable tools for fluorescence imaging technology and significantly impact the ultimate quality of imaging. The development of imaging technology has continued to impose higher requirements on fluorescent dyes, and this has promoted the improvement and optimization of the properties of the dyes. The development of super-resolution fluorescence microscopy has broken the optical diffraction limit and made it possible to study the fine structure of various cellular organelles using fluorescence microscopy. More and more researchers have begun to use super-resolution fluorescence microscopy to study the interactions of subcellular structures and have achieved many groundbreaking advancements. However, achieving super-resolution imaging of live cells imposes stringent demands on various optical properties of fluorescent dyes. The ideal fluorescent dye for live cell super-resolution imaging should possess excellent photostability, a high fluorescence quantum yield, good water solubility, the ability to meet the specific requirements of super-resolution systems (such as spontaneously blinking or quenching under depletion light), and also excellent membrane permeability.

To address these problems, chemists have developed a number of new theories, such as the lactone-zwitterion equilibrium constant (*K*_L-Z_) theory, to make fluorescent dyes maximally satisfy the above requirements. In addition, researchers have introduced a number of methods to increase the membrane permeability of existing super-resolution probes, thereby expanding the options available for probes used in live-cell imaging and facilitating the development of multicolor live cell super-resolution fluorescence microscopy. Combining these probes with super-resolution imaging techniques, researchers have gained a deeper understanding of the dynamic interactions of organelles. However, compared with the range of resources available for traditional fluorescence imaging, there are still few probes available for live cell super-resolution imaging. In addition, in recent years, live cell super-resolution imaging technology has gradually developed from 2D imaging to 3D imaging, which further reduces the necessary exposure time for ensuring temporal resolution, thus posing higher demands on the fluorescence properties of fluorescent dyes [97–99]. To design fluorescent dyes with even better fluorescence properties, our understanding of the fluorescence mechanism of fluorescent dyes needs to be improved. In recent years, artificial intelligence (AI) technology has been widely used in molecular drug design, and this has significantly advanced the development of chemical drugs; the use of AI-assisted design of novel fluorescent groups is expected to further enhance the fluorescence properties of fluorescent dyes. The design of live-cell probes often requires some compromise in fluorescence performance to balance membrane permeability. In contrast, probes designed in combination with the cell-impermeable probe live-cell delivery technology are not dependent on their cell permeability, and this can further enhance the fluorescence performance of the probes. The fluorescence brightness of live-cell probes is expected to be further enhanced by integrating these technologies. However, the existing technologies for live-cell delivery of cell-impermeable probes are more demanding for cell types, and the labeling methods are more complicated than those for cell-permeable probes, making such technologies difficult to widely disseminate [92]. Therefore, it is necessary to further develop cell-impermeable probe live cell delivery strategies with low toxicity and high delivery efficiency. The development of these high-performance probes will further promote the development of cell biology.

## Data Availability

All data are provided in the article.
